# Advantage of Tolerability following Arsenic Trioxide-VTD vs VRD in newly diagnosed multiple myeloma patients: a prospective, open-label study

**DOI:** 10.7150/ijms.110231

**Published:** 2025-04-28

**Authors:** Xinyu Zuo, Apeng Yang, Pingping Chen, Yanhui Xie, Zhiyong Zeng, Jiexian Ma

**Affiliations:** 1 Department of Hematology, Huadong Hospital, Fudan University, Shanghai, China.; 2 Department of Hematology, The First Affiliated Hospital of Fujian Medical University, Fuzhou, China.

**Keywords:** multiple myeloma, arsenic trioxide, tolerability, treatment response, cost-effectiveness

## Abstract

Multiple myeloma is the second most common hematologic malignancy in older patients. The standard front-line VRD regimen (bortezomib/lenalidomide/dexamethasone) achieves high efficacy but is associated with significant toxicity, leading to infections, bone marrow suppression, and treatment discontinuation in approximately 20% of patients. Alternative regimens with reduced toxicity are needed for this demographic. Prior studies suggest adding arsenic trioxide to bortezomib/dexamethasone (BD) enhances remission depth with acceptable safety, while bortezomib/thalidomide/dexamethasone (VTD) offers reduced toxicity, but lower efficacy compared to VRD.

This study evaluates the efficacy, safety, and cost-effectiveness of an arsenic trioxide-VTD regimen (AVTD) versus VRD in newly diagnosed multiple myeloma (NDMM) patients. Among 116 participants, AVTD demonstrated comparable efficacy to VRD but significantly reduced infection rates (14.0% vs. 40.7%, P < 0.001) and severe bone marrow suppression (0% vs. 11.9%, P = 0.013). Subgroup analysis of patients >60 years yielded consistent results. Additionally, AVTD was associated with lower treatment costs.

In conclusion, the AVTD regimen offers a safer, more cost-effective alternative to VRD for NDMM, particularly in older adult patients, without compromising treatment efficacy.

## Introduction

Multiple myeloma (MM) is a malignancy characterized by clonal plasma cell proliferation. It stands as the second most prevalent malignant tumor within the hematological system 1. Worldwide the disease sees over 588,000 new diagnoses annually, with nearly 100,000 new deaths 2. The financial burden of MM is also significant; after commencing anti-myeloma treatments, the average annual adjusted costs surpass $110,000, ranking it among the most costly cancers to manage 3-5. In 2016, China reported an overall incidence rate of MM at 1.03/100,000 and a mortality rate of 0.67/100,000, both of which have been on a steady rise 6. Notably, two-thirds of MM patients are 65 years or older 7. Given the global trend towards an aging population, the number of MM patients is expected to rise, and the physiological changes accompanying aging can diminish a patient's treatment tolerance. Adverse events related to treatments leading to interruptions or discontinuations are significant contributors to unfavorable prognosis 8.

The combination of bortezomib, lenalidomide, and dexamethasone (VRD) is the standard front-line treatment for newly diagnosed multiple myeloma (NDMM). However, its use in older patients is associated with a heightened risk of adverse reactions 9. Common adverse reactions, such as bone marrow suppression (47%), neurotoxicity (33%), fatigue (16%) and infections (14.5%) have been reported. Consequently, between 17.2% and 22.6% of patients discontinue the medication due to these adverse effects and subsequent relapses 9-12. Thus, there is a pressing need, especially for older patients, to devise a treatment strategy that strikes a balance between efficacy and safety. Addressing the economic implications of prolonged treatment is also of paramount socio-economic importance 13, it will also be a pivotal research area in myeloma management to minimize treatment suspensions due to adverse reactions.

Arsenic acid first demonstrated its therapeutic potential in China, where it was effectively used to treat acute promyelocytic leukemia 14. Following this success, the drug was explored in preclinical and early clinical trials for other malignancies, including MM 15. Both theoretical analyses and clinical trials have indicated that arsenic trioxide exhibits potent anti-myeloma effects, either as a stand-alone treatment or in combination with other anti-cancer agents, like MAC regimen (melphalan/arsenic trioxide/Vitamin C) and ABC regimen (arsenic trioxide/bortezomib/ascorbic acid) 16-19. Our prior clinical research has established that for NDMM patients, the combination of arsenic trioxide with the bortezomib/dexamethasone regimen offers superior safety and efficacy compared to the bortezomib/dexamethasone (BD) regimen alone 20.

In MM therapy, bortezomib/thalidomide/dexamethasone (VTD) regimen remains a valuable treatment option for older adult patients, while VTD showed weaker efficacy over the first-line VRD regimen 21. Integrating arsenic acid with the VTD regimen might further amplify VTD's therapeutic benefits without significantly elevating its toxicity profile. Yet, it remains uncertain whether the combination of arsenic trioxide and VTD offers any advantages over the standard first-line MM treatment, VRD. To address this, we compared the arsenic trioxide/bortezomib/thalidomide/dexamethasone (AVTD) regimen with the VRD regimen in treating newly diagnosed MM patients in a prospective study. This clinical trial was conducted across two medical centers: Huadong Hospital Affiliated with Fudan University and the First Hospital Affiliated with Fujian Medical University. We enrolled a total of 116 patients, with median age above 60 years. Our objective was to discern differences in efficacy, safety, and treatment costs between the AVTD and VRD regimens in newly diagnosed myeloma patients, especially the older adult patients.

## Methods

### Patients

This study was a prospective, open-label trials conducted at 2 centers in China. Between January 2022 and January 2024, 116 patients from Huadong Hospital and The First Hospital Affiliated with Fujian Medical University were enrolled. Eligible participants for this study were newly diagnosed MM patients with measurable serum and/or urine M protein. Key inclusion criteria included: age ≥18 years; a new MM diagnosis; a Zubrod performance status score of <4; no prior treatment with arsenic therapy; a left ventricular ejection fraction >40%; absence of uncontrolled arrhythmia or unstable cardiac conditions; a corrected QT interval <470 ms; no symptomatic pulmonary conditions with satisfactory pulmonary function tests; serum glutamic pyruvic transaminase levels <4 × the upper limit of normal; serum bilirubin levels <2 × the upper limit of normal; and a performance status <3. Major exclusion criteria encompassed peripheral neuropathy grade ≥2, systemic amyloidosis, and a positive serology for HIV (and HIV-1, HIV-2) or hepatitis B or C. The Huadong Hospital's institutional ethics committee approved the study (2022K117), and it adhered to the principles of the Declaration of Helsinki. All participants provided written informed consent. The study was registered in the Chinese Clinical Trials Registry (Number ChiCTR2400083240).

### Study Design and Endpoints

Patients enrolled into this study were randomly divided into two groups receiving AVTD regimen or VRD regimen. The AVTD regimen for patients was as follows: arsenic trioxide 0.16 mg/kg on days 1-3, 8-10, and 15-17 over 2 hours; thalidomide 100mg on days 1-21; Bortezomib 1.6 mg/m^2^ subcutaneously on days 4, 11, and 18; and dexamethasone 40 mg/day IV on days 4-7, 11-14, and 18-21. For patients over 65 years or those with diabetes, dexamethasone dosage was reduced to 20 mg/day. This regimen was repeated every 28 days. The VRD regimen comprised four 21-day cycles Bortezomib was given at 1.3 mg/mg^2^ intravenously on days 1, 4, 8 and 11, combined with oral lenalidomide 25 mg daily on days 1-14 plus oral dexamethasone 20 mg daily on days 1, 2, 4, 5, 8, 9, 11, and 12 9. Supportive care was provided as per departmental guidelines. Patients received granulocyte colony-stimulating factor 5 μg/kg/day if the absolute neutrophil count dropped below 0.5 ×10^9^/L for two consecutive days. Prophylactic oral levofloxacin, acyclovir, and fluconazole were administered during neutropenia. Blood products were given if hemoglobin levels were <6 g/dL or platelet counts were <20 × 10^9^/L. The induction therapy duration was consistent at 16 weeks across all arms. Patients on lenalidomide induction therapy were recommended either low-molecular-weight heparin or aspirin thromboprophylaxis. After two and four cycles of chemotherapy respectively, each patient's disease status was evaluated. Those with stable disease (SD) or progressive disease (PD) after two cycles were transitioned to alternative regimens. The primary endpoint was the treatment response, with safety being the secondary endpoint.

### FISH Studies

Bone marrow plasma cells were isolated using anti-CD138-coated magnetic beads via the AutoMACs automated separation system (Miltenyi Biotec). Interphase fluorescence *in situ* hybridization (FISH) was conducted using specific probes (Abbott Molecular/Vysis) targeting 17p deletions, as well as immunoglobulin heavy chain translocations, including t(4;14) and t(14;16), and 1q21, as previously detailed 22. All cytogenetic evaluations were centrally conducted at the Huadong Hospital.

### Response Criteria

Patient responses were assessed at the onset of each treatment cycle, following standard International Myeloma Working Group (IMWG) response criteria.^23^ Safety was monitored for 30 days post the final drug dose.

### Safety

Adverse events were categorized based on the National Cancer Institute Common Toxicity Criteria (Version 5.0) 24. Bortezomib or combination chemotherapy was withheld in instances of grade 4 hematologic toxicity or grade ≥3 nonhematologic toxicity until the toxicity subsided to grade ≤2. For bortezomib-related toxicities, once resolved, the drug was reintroduced at a 25% reduced dose. Management of bortezomib-induced peripheral neuropathy or neuropathic pain followed established protocols 25,26. Thalidomide-related peripheral neuropathy of grade 2 led to a 50% dose reduction, while grade 3 necessitated discontinuation until symptoms reduced to grade ≤ 1, followed by a 50% dose reintroduction. For dexamethasone-related grade 3 or 4 adverse events, the drug was withheld until toxicity subsided to grade 2 or less, followed by a 50% dose reduction. Lenalidomide was discontinued in cases of extensive rash. With dosage adjustments as necessary using slide adjustment scale within the VRD protocol.

### Costs

This study considered direct medical costs, encompassing drug expenses. Related medical costs included hospitalization, laboratory tests, diagnostic procedures, concomitant treatments, hospital visits, and treatment costs for severe adverse reactions. The pricing for lenalidomide, thalidomide, arsenic trioxide, and bortezomib was based on the medical insurance payment standards negotiated by the China National Medical Security Administration. Dexamethasone pricing followed the tender prices in Shanghai City and Fujian Province. In our study, all costs were updated to March 2024 US dollars by the United States Consumer Price Index.

### Disease Monitoring

Disease assessments, which included skeletal surveys, complete neurologic examinations, Karnofsky performance status evaluations, beta2-microglobulin, C-reactive protein, serum and urine electrophoresis for immunoglobulin quantification, immunofixation, bone marrow aspiration, and biopsy, were conducted within 14 days prior to the first day of the initial treatment cycle. Comprehensive medical histories were collected, and baseline physical and complete neurologic examinations were performed. Additionally, 12-lead electrocardiography and posteroanterior and lateral chest X-rays were taken. Bone marrow aspirates were evaluated, and biopsies were supplemented with flow cytometry, chromosome analysis and FISH. Clinical laboratory tests, including hematology, clinical chemistry, electrolyte and glucose panels, total protein, amylase, albumin tests, urinalysis, and serum pregnancy tests for women of child-bearing potential, were conducted on the first day of each cycle. Treatment assessment staging was initiated at entry, with restaging after two cycle and four cycles. Three authors independently collected patient medical data to minimize bias. Extracted patient information included age, sex, disease stage, blood cell count, treatment response, and survival status. No significant selection bias is anticipated in these clinical data collections and analyses.

### Statistical Analyses

Patient characteristics were summarized using means and 95% confidence intervals (CI) for numerical variables and frequencies with percentages for categorical variables. Differences between the two treatment groups were assessed using two-sample t-tests or Mann-Whitney U-test for numerical variables and Chi-squared tests or Fisher's exact test for categorical variables. Logistic regression was employed to analyze factors influencing treatment response. To enhance statistical efficiency and mitigate confounding factors, patients in two treatment regimens were matched in terms of age ± 5 (year) as a group matching at a 1:1 ratio. All statistical analyses were performed using SPSS version 27.0 for Windows (IBM SPSS, Chicago, IL, USA) with statistical significance defined as two-sided and P-values < 0.05.

## Results

### Clinical Characteristics of the Two Treatment Groups

**Figure 1:**
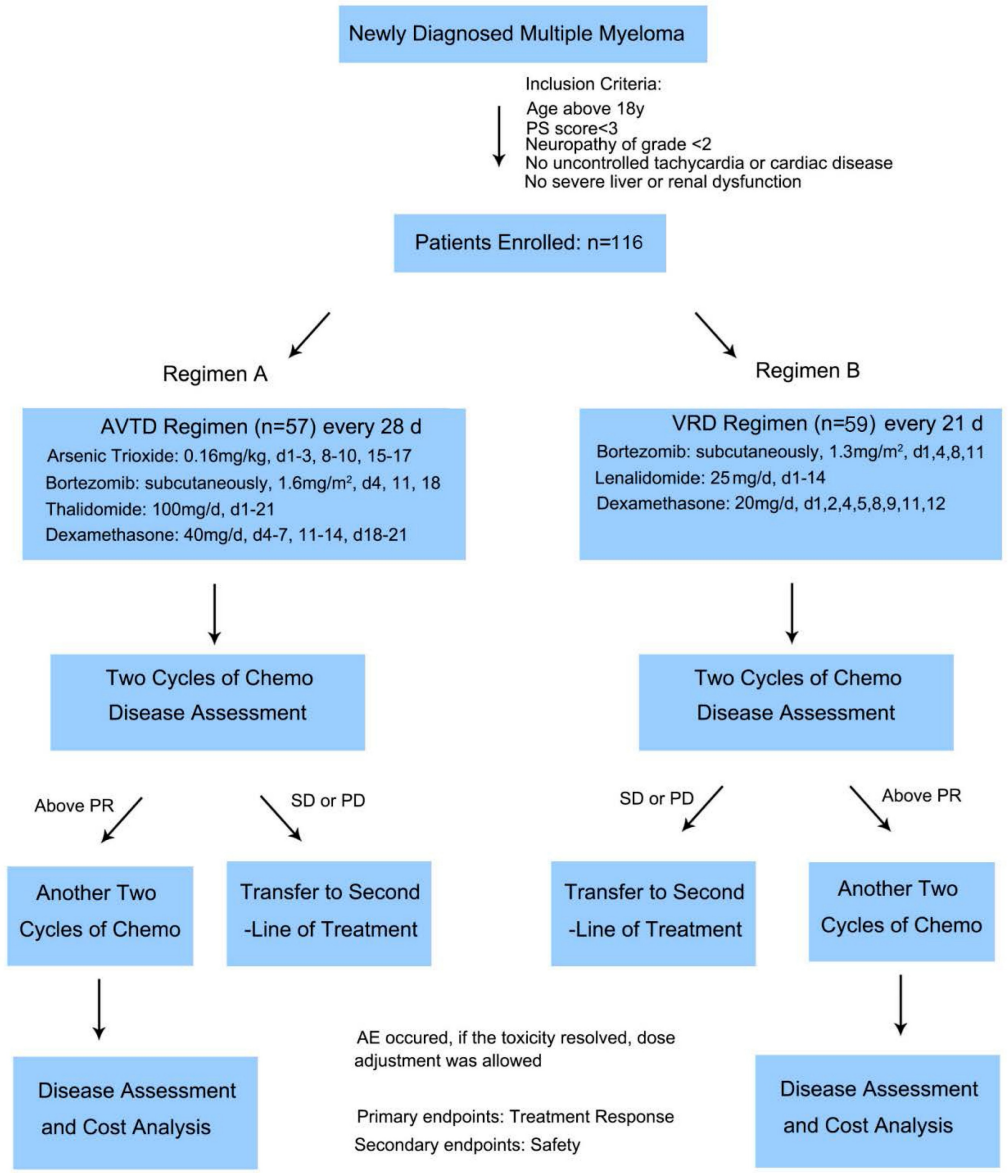
The Flowchart of the study and the protocols of the two regimens

Of the 116 enrolled and treated patients, 57 received the AVTD regimen and 59 underwent the VRD regimen. The Flowchart of the study was shown in Figure 1, Table 1 displays the pretreatment characteristics of patients based on their treatment arm. Prognostic factors, such as ISS stage and cytogenetic status, were evenly distributed between the two treatment groups. The median age for both groups exceeded 60 years. Factors like gender, isotype, Durie-Salmon Stage at diagnosis, ISS stage at diagnosis, cytogenetic abnormalities, Lactate Dehydrogenase (LDH) level, albumin level, and beta2-microglobulin level at diagnosis exhibited no significant disparities between the two treatment groups. However, the VRD group had a higher incidence of del(17/17p) (11.9% vs 0%, P=0.013), while the AVTD group had a higher incidence of t(4,14) (28.1% vs 8.5%, P=0.008). No significant differences were observed between the two groups regarding high-risk subtypes detected by FISH according to Mayo mSMART risk stratification^22^ (P=0.460).

### Treatment Response, Safety, and Tolerability to the Induction Therapy

All patients completed the planned therapy sequence, were evaluable for response, and underwent comprehensive assessment. Table 2 summarizes the response rates. The clinical response between the AVTD and VRD groups did not differ significantly (P=0.268). The percentage of patients with a better response (sCR + CR + VGPR) in the AVTD group was higher than that in the VRD group, but the difference was not statistically significant (75.4% vs 62.7%, P=0.139). Adverse effects and their frequencies are detailed in Table 3. All adverse effects were manageable with appropriate treatment, and no fatalities were attributed to these effects. The AVTD group exhibited a lower incidence of any grade AEs related to hematologic toxicity. The incidence of Grade Ⅰ to Ⅱ blood hypocellular was lower in AVTD group (3.5% vs 17.2%, P=0.015) compared to VRD group, as well as the grade III to IV blood hypocellarity (0% vs 12.5%, P=0.006). Common infection was in respiratory system (14.0% vs 47.5%, P<0.001) and febrile neutropenia (0 vs 13.6%, P=0.004). Any grade AEs (pain or neuritis) in neurological (Grade I to II:15.8% vs 35.6%, P=0.015) is lower compared to the VRD group. Grade III to IV neuritis was scarcely seen in both groups (Table 3).

### Treatment Response, Safety, and Tolerability to the Induction Therapy in Patients Above 60 Years Old

To ascertain the efficacy and safety of the AVTD regimen for older adult patients, we segregated the two treatment groups based on age (above or below 60 years). We then compared the clinical characteristics (Table S1), treatment response (Table S2), and adverse effects (Table S3) of the subgroup aged over 60 years. The results revealed no significant statistical differences between the AVTD and VRD groups in terms of clinical characteristics. For older adult patients, the deep response rate (sCR+CR) and treatment response rate (sCR+CR+VGPR) between the AVTD and VRD regimens were comparable (70% vs 71.4%, P=0.9, Table S2). The AVTD group had a lower incidence of infection in lung or upper respiratory, (20.0% vs 57.1%, P=0.002) and febrile neutropenia (0 vs 14.3%, P=0.021) with less hematologic toxicity compared to the VRD group. Lower incidence of Grade I to II neuropathy (16.7% vs 40.0%, P=0.036, Table S3). None patients in the study experienced severe neuropathy leading to treatment interruption or discontinuation. The neuropathy was mild to moderate and tolerable, only 5% patients in VRD group suffered Grade III to IV neuritis.

### Independent Prognostic Factor of the AVTD Regimen and Age-matched Paired Chi-Square Test of the Two Regimens

**Figure 2:**
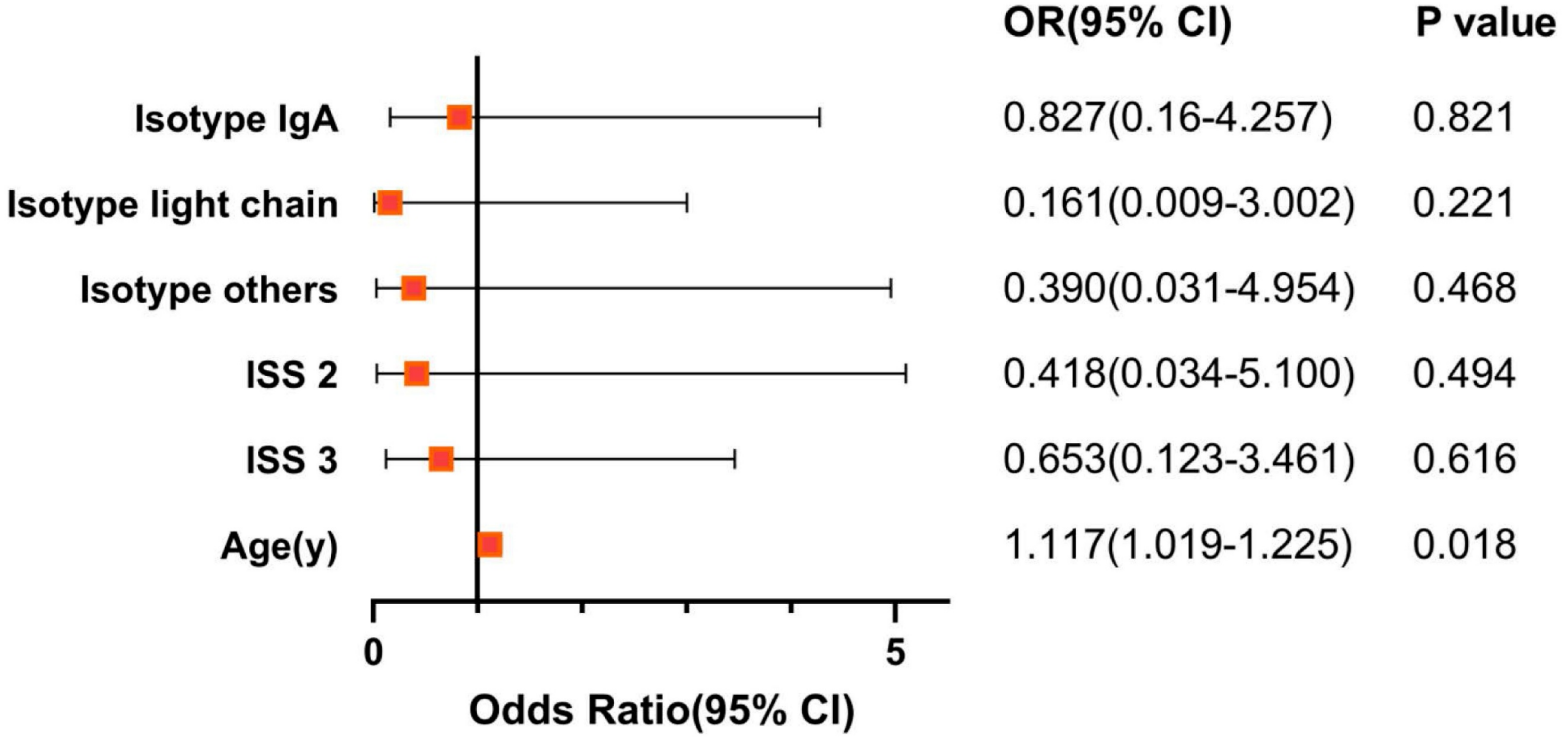
The Forest Plot of prognostic factor on AVTD regimen

To identify independent prognostic factors influencing the efficacy of the AVTD regimen, both univariate and multivariate logistic regression analyses were performed specifically on the AVTD group. Multivariate regression analysis indicated that age might serve as an independent prognostic factor for the AVTD regimen, as depicted in Figure 2. To enhance the precision of the statistical analysis and mitigate the bias introduced by age, we paired patients from the AVTD and VTD regimens in a 1:1 ratio, ensuring an age difference of no more than 5 years between matched pairs. Post-matching, 23 pairs were identified, totaling 46 patients. Subsequent paired chi-square analysis, as presented in Table S4, reaffirmed the earlier conclusion that the efficacy of the AVTD regimen is comparable to that of the VRD regimen (sCR+CR+VGPR: 76.1% vs 58.7%, P=0.134). Regarding adverse reactions, the AVTD regimen exhibited a significantly lower incidence of grade III to IV bone marrow suppression (0% vs 13.0%, P=0.011) and infections in respiratory system (15.2% vs 50.0%, P<0.001), as well as febrile neutropenia (0 vs 15.2%, P=0.006).

### Economic Cost Comparison between the AVTD and VRD Regimens

Costs were categorized into three segments: in-hospital expenses (Total cost), direct drug costs (expenses related to in-hospital and outpatient myeloma treatments), and treatment-related costs (expenses associated with complications induced by the disease and its treatment, such as infections). Across all categories, the AVTD regimen was found to be significantly more cost-effective than the VRD regimen (P<0.01, Table 4). This conclusion held true both for the subgroup aged above 60 years and in the age-matched paired analysis. The cost of treatment was reduced either by drugs themselves or by the supportive medical treatment for complications such as infections and pancytopenia.

## Discussion

Our findings indicate that for newly diagnosed multiple myeloma patients, particularly the older adult patients, the AVTD regimen demonstrates comparable efficacy to the VRD regimen in terms of inducing remission. AVTD regimen mirrors the therapeutic effects of the VRD regimen as well markedly diminishes the risks of bone marrow suppression, infections and neuropathy. Additionally, AVTD regimen substantially alleviates the long-term economic burden on patients. The likelihood of halting treatment is also minimized, ensuring optimal disease management and enhancing patients' outcomes. Given its potent efficacy coupled with reduced toxicity, this regimen may be particularly beneficial for the older adult patients.

With the advent of proteasome inhibitors and immunomodulatory drugs, there have been significant advancements on prognosis in newly diagnosed multiple myeloma, notably in the older adult patients 27. Daratumumab, a human IgGκ monoclonal antibody that targets CD38, combined with lenalidomide and dexamethasone, reduces disease progression or mortality risk compared to lenalidomide and dexamethasone alone but has higher incidence of neutropenia and pneumonia 28. Beyond disease control, life quality is of paramount importance. The AVTD regimen, through addition of arsenic trioxide with anti-tumor agents like bortezomib, thalidomide, and dexamethasone, has demonstrated promising outcomes and safety in treating multiple myeloma.

We postulate that the diminished incidence of adverse reactions, such as infections and bone marrow suppression in the AVTD regimen, can be attributed to the following reasons: lenalidomide is known to exhibit greater bone marrow suppressive toxicity compared to thalidomide 29. Additionally, arsenic trioxide typically induces minimal adverse reactions and is rarely associated with bone marrow suppression and infection in patients 30. Further, Arsenic acid might booster specific and non-specific immune responses against MM cells by modulating antigen-presenting function of dendritic cells (DC), NK cells 31 and inhibiting the production of IL-6 and VEGF 32,33 to overcome the immune-compromised state in MM 34.

The observed efficacy of the AVTD regimen, comparable to that of the VRD regimen, may be attributed to the synergistic effects of arsenite combined with bortezomib, thalidomide, and dexamethasone. Interleukin-6 (IL-6), a known growth factor for MM 35, promote the tumor progression via activating the JAK-STAT3 pathway 36, facilitating the adhesion of myeloma cells to bone marrow mesenchymal stem cells 37 and obstructing dexamethasone-induced apoptosis 38. Arsenic acid has been shown to inhibit STAT3 activation in myeloma cells 39, consequently reducing IL-6 release. Arsenite also has synergy with dexamethasone to induce MM cell apoptosis through the Caspase-9 signaling pathway 40. Arsenic trioxide can diminish key effector proteins in the classical Wnt signaling pathway, reducing β-Catenin accumulation, which results in inhibited myeloma cell proliferation 41, increased apoptotic cell proportions, and heightened sensitivity of myeloma cells to bortezomib 42.

The value of AVTD regimen's value, especially for older adult patients, is evident given its reduced adverse reactions, outstanding tolerability, and potent therapeutic response. Does this regimen make any sense in younger patients? An intriguing observation, which we didn't heavily underscore in our results, emerges from logistic regression analysis. Age appears to be an independent prognostic factor for AVTD, suggesting that younger patients might exhibit a more favorable treatment response. The treatment response rate (sCR+CR+VGPR) of the AVTD regimen surpassed that of the VRD regimen in patients under 60 years old, which might due to arsenite exerts its anti-tumor effects by increased production of reactive oxygen species (ROS). ROS can trigger a cascade of events culminating in cell apoptosis 43. Studies have indicated that advanced age confers a protective effect on oxidative stress 44. Consequently, compared to older patients, an elevation in ROS levels may have a more pronounced impact on myeloma cells in younger individuals.

With age, there's a consistent rise in incidence and mortality rates 45. Hence, for the older adult patients, the AVTD regimen, being safer, more effective, and cost-efficient, warrants deeper investigation and further research to discern the underlying mechanism. And the data we currently have are limited and definitive conclusions remain elusive. Future prospective, multicenter clinical trials may provide greater clarity.

## Supplementary Material

Supplementary tables.

## Figures and Tables

**Table 1:** Baseline Clinical Characteristics of Patients Receiving the Two Regimens

Characteristics	AVTD(n=57)	VRD (n=59)	p value
**Age, years**			0.399
Median (range)	60(47-84)	62(46-81)	
**Gender, n (%)**			0.922
Male	30(52.6)	31(52.5)	
**Myeloma type, n (%)**			0.232
Immunoglobulin G	25(43.9)	33(58.9)	
Immunoglobulin A	13(22.8)	13(23.2)	
Light chain disease only	13(22.8)	8(14.3)	
Others	6(10.5)	2(3.6)	
**Durie-Salmon stage at diagnosis, n (%)**			0.088
I	5(8.8)	9(15.3)	
II	12(21.1)	5(8.5)	
III	40(70.2)	45(76.3)	
**International Staging System stage at diagnosis, n (%)**			0.139
I	10(17.5)	17(28.8)	
II	20(35.1)	24(40.7)	
III	27(47.4)	18(30.5)	
**Cytogenetic abnormalities determined by FISH, n (%)**			
del(17/17p)	0(0)	7(11.9)	0.013
t(4,14)	16(28.1)	5(8.5)	0.008
t(14,16)	1(1.8)	1(1.7)	1.000
1q21	19(33.3)	18(30.5)	0.744
High risk 22	36(63.2)	31(52.5)	0.460
**LDH**			0.589
Mean, 95% CI	204.15 (166.68-241.62)	222.34 (168.23-276.46)	
**Median beta2-microglobuli, mg/L, n (%)**			0.183
<3.5	20(35.1)	25(42.4)	
≥3.5, <5.5	14(24.6)	19(32.2)	
≥5.5	23(40.4)	15(25.4)	
**Albumin**			0.297
Mean, 95% CI	34.46 (32.41-36.52)	38.24 (31.83-44.64)	

**Table 2:** Treatment Response (After Four Cycles)

	AVTD		VRD		p value
	n	%	n	%	
Response					0.268
sCR	2	3.5	5	8.5	
CR	13	22.8	13	22.0	
VGPR	28	49.1	19	32.2	
PR	11	19.3	15	25.4	
PD	3	5.3	7	11.9	
sCR+CR	15	26.3	18	30.5	0.617
sCR+CR+VGPR	43	75.4	37	62.7	0.139
Above PR	54	94.7	52	88.1	0.322

**Table 3:** Comparison of Adverse Effects Associated with the Two Regimens

	ATVD (n=57)	VRD (n=59)	p value
**Hematological**			
Grade1-2 Blood or bone marrow hypocellarity, n (%)	2(3.5)	10(16.9)	0.029
Grade3-4 Blood or bone marrow hypocellarity, n (%)	0	7(11.9)	0.013
**Infection**			
Febrile neutropenia, n (%)	0	8(13.6)	0.004
Lung or upper respiratory infection, n (%)	8(14.0)	28(47.5)	<0.001
Sepsis, n (%)	0	2(3.4)	0.161
**Neurological**			
Grade 1-2 Pain or neuritis, n (%)	9(15.8)	21(35.6)	0.015
Grade 3-4 Pain or neuritis, n (%)	0	2(3.4)	0.496
**Non-hematological or non-neurological**			
Diarrhea, n (%)	6(10.5)	1(1.7)	0.059
Edema, n (%)	5(8.8)	2(3.4)	0.268
Constipation, n (%)	2(3.5)	5(8.5)	0.439
Hyperkalemia, n (%)	7(12.3)	14(23.7)	0.148
Tachycardia or prolonged QT interval, n (%)	8(14.0)	5(8.5)	0.390

**Table 4:** The Cost of Treatment in the Two Treatment Regimens

	AVTD vs VRD	p value
**All patients**	**n=57 vs n=59**	
Total cost of each treatment cycles, USD (mean, 95% CI)	5633.42(5366.46-5900.38) vs 11184.89(9856.31-12513.74)	<0.001
Direct drug cost, USD (mean, 95% CI)	3003.44(2861.79-3145.08) vs 6481.30(5919.85-7042.75)	<0.001
Treatment related cost, USD (mean, 95% CI)	2735.32(2657.52-2813.12) vs 4629.59(4091.56-5761.61)	<0.001
**Subgroup of Age above 60 y**	**n=30 vs n=35**	
Total cost of each treatment cycles, USD (mean, 95% CI)	5846.29(5513.04-6179.53) vs 11309.54 (9526.12-13079.31)	<0.001
Direct drug cost, USD (mean, 95% CI)	3089.49(2850.60-3328.37) vs 6590.41 (5871.05-7309.76)	<0.001
Treatment related cost, USD (mean, 95% CI)	2756.80(2638.64-2874.96) vs 4997.96 (3934.23-6061.68)	<0.001
**Age matched analysis**	**23 pairs**	
Total cost of each treatment cycles, USD (mean, 95% CI)	5759.14(5518.76-5999.52) vs 10374.12(9076.41-11671.84)	<0.001
Direct drug cost, USD (mean, 95% CI)	3028.29(2858.73-3197.85) vs 6130.26 (5586.82-6673.69)	<0.001
Treatment related cost, USD (mean, 95% CI)	2727.91(2638.52-2817.30) vs 4571.87(3746.71-5397.02)	<0.001

## Abbreviations

VRD: bortezomib/lenalidomide/dexamethasone
BD: bortezomib/dexamethasone
VTD: bortezomib/thalidomide/dexamethasone
NDMM: newly diagnosed multiple myeloma
AVTD: arsenic trioxide/bortezomib/thalidomide/dexamethasone
MM: multiple myeloma
HIV: human immunodeficiency virus
SD: stable disease
PD: progressive disease
FISH: Fluorescence *in situ* hybridization
IMWG: International Myeloma Working Group
CI: confidence intervals
LDH: Lactate Dehydrogenase
ROS: reactive oxygen species
