# Supplementary material for: Advantage of Tolerability following Arsenic Trioxide-VTD vs VRD in newly diagnosed multiple myeloma patients: a prospective, open-label study
Source: Int J Med Sci. 2025 Apr 28;22(10):2373–81. doi: 10.7150/ijms.110231 (PMC12080582; doi:10.7150/ijms.110231)
Supplement: Supplementary file 1 — Supplementary tables. [file ijmsv22p2373s1.pdf]

Supplemental Materials

Table S1. Baseline Clinical Characteristics of Patients in Subgroup (Age≥60)

Characteristics	AVTD(n=30)	VRD(n=35)	p value
**Age, years**			0.680
Median (range)	64(60-84)	67(60-81)	
**Gender, n (%)**			
Male	14(46.7)	21(60.0)	0.282
**Myeloma type, n (%)**			0.127
Immunoglobulin G	12(40.0)	22(68.8)	
Immunoglobulin A	10(33.3)	7(21.9)	
Light chain disease only	5(16.7)	2(6.3)	
Others	3(10.0)	1(3.1)	
**Durie-Salmon stage at diagnosis, n (%)**			0.306
I	5(16.7)	5(14.3)	
II	9(30.0)	5(14.3)	
III	16(53.3)	25(71.4)	
**International Staging System stage at diagnosis, n (%)**			0.664
I	7(23.3)	6(17.1)	
II	14(46.7)	15(42.9)	
III	9(30.0)	14(40.0)	
**Cytogenetic abnormalities determined by FISH, n (%)**			
del(17/17p)	0(0)	6(17.1)	0.027
t(4,14)	7(23.3)	4(11.4)	0.320
t(14,16)	1(3.3)	0(0)	0.462
1q21	10(33.3)	7(20.0)	0.223
High risk[22]	18(60.0)	17(42.5)	0.654
**LDH**			0.864
Mean, 95% CI	188.55(160.38-216.71)	237.76(150.32-325.18)	
**Median beta2-microglobuli, mg/L,n(%)**			0.439
<3.5	12(40.0)	12(34.3)	
≥3.5, <5.5	7(23.3)	13(37.1)	
≥5.5	11(36.7)	10(28.5)	
**Albumin**			0.345
Mean, 95% CI	34.83(31.48-37.98)	38.62(28.13-49.12)	

Table S2. Treatment Response (After Four Cycles) in Patients in Subgroup (Age≥60)

	AVTD		VRD		p value
	n	%	n	%	
**Response**					0.715
sCR	1	3.3	3	8.6	
CR	5	16.7	9	25.7	
VGPR	15	50.0	13	37.1	
PR	7	23.3	7	20.0	
PD	2	6.7	3	8.6	
sCR+CR	6	20.0	12	34.3	0.199
sCR+CR+VGPR	21	70.0	25	71.4	0.900
Above PR	28	93.3	32	91.4	0.100

Table S3. Comparison of Adverse Effects Associated with the Two Regimens in Patients in Subgroup (Age≥60)

	ATVD(n=30)	VRD(n=35)	p value
**Hematological**			
Grade1-2 Blood or bone marrow hypocellular, n (%)	1(3.3)	8(22.9)	0.023
Grade3-4 Blood or bone marrow hypocellular, n (%)	0	5(14.3)	0.031
**Infection**			
Febrile neutropenia, n (%)	0	5(14.3)	0.031
Lung or upper respiratory infection, n (%)	6(20.0)	20(57.1)	0.002
Sepsis, n (%)	0	1(2.9)	1.000
**Neurological**			
Grade 1-2 Pain or neuritis, n (%)	5(16.7)	14(40.0)	0.036
Grade 3-4 Pain or neuritis, n (%)	0	2(5.7)	0.495
**Non-hematological or non-neurological**			
Diarrhea, n (%)	3(10.0)	0(0)	0.093
Edema, n (%)	2(6.7)	1(2.9)	0.591
Constipation, n (%)	2(6.7)	2(5.7)	0.100
Hyperkalemia, n (%)	4(13.3)	10(28.6)	0.226
Tachycardia or prolonged QT interval, n (%)	5(16.7)	1(2.9)	0.087

Table S4. Age Paired Chi-square Analysis in the Two Regimens

	ATVD	VRD	p value
**Treatment Response**			

sCR+CR,n(%)	12(26.1)	14(30.4)	0.815
sCR+CR+VGPR,n(%)	35(76.1)	27(58.7)	0.134
Above PR,n(%)	44(95.7)	39(84.8)	0.180
**Adverse events**			
**Hematological**			
Grade1-2 Blood or bone marrow hypocellular, n (%)	1(2.2)	5(10.9)	0.203
Grade3-4 Blood or bone marrow hypocellular, n (%)	0	6(13.0)	0.011
**Infection**			
Febrile neutropenia, n (%)	0	7(15.2)	0.006
Lung or upper respiratory infection, n (%)	7(15.2)	23(50.0)	<0.001
Sepsis, n (%)	0	1(2.2)	1.000
**Neurological**			
Grade 1-2 Pain or neuritis, n (%)	6(13.0)	14(30.4)	0.043
Grade 3-4 Pain or neuritis, n (%)	0	1(2.2)	1.000
**Non-hematological or non-neurological**			
Diarrhea, n (%)	5(10.9)	2(4.3)	0.453
Edema, n (%)	4(8.7)	2(4.3)	0.687
Constipation, n (%)	4(8.7)	6(13.0)	0.754
Hyperkalemia, n (%)	6(13.0)	11(23.9)	0.302
Tachycardia or prolonged QT interval, n (%)	6(13.0)	4(8.7)	0.739
